# Identifying Recent HIV Infections: From Serological Assays to Genomics

**DOI:** 10.3390/v7102887

**Published:** 2015-10-23

**Authors:** Sikhulile Moyo, Eduan Wilkinson, Vladimir Novitsky, Alain Vandormael, Simani Gaseitsiwe, Max Essex, Susan Engelbrecht, Tulio de Oliveira

**Affiliations:** 1 Division of Medical Virology, Stellenbosch University, Tygerberg 7505, South Africa; susanen@sun.ac.za; 2 Botswana-Harvard AIDS Institute Partnership, Private Bag BO320, Gaborone, Botswana; vnovi@hsph.harvard.edu (V.N.); sgaseitsiwe@bhp.org.bw (S.G.); messex@hsph.harvard.edu (M.E.); 3 Africa Centre for Health and Population Studies, Doris Duke Medical Research Centre, Nelson R Mandela School of Medicine, University of KwaZulu-Natal, Durban 4041, South Africa; ewilkinson83@gmail.com@email (E.W.); vando026@umn.edu (A.V.); 4 Department of Immunology and Infectious Diseases, Harvard T.H. Chan School of Public Health, Boston, MA 02115, USA; 5 National Health Laboratory Services (NHLS), Tygerberg Coastal, Johannesburg 2001, South Africa; 6 Research Department of Infection, University College London, London WC1E 6BT, UK; 7 College of Health Sciences, University of KwaZulu-Natal, Durban 4041, South Africa

**Keywords:** recent HIV infection, viral diversity, serology-based assays, molecular-based assays

## Abstract

In this paper, we review serological and molecular based methods to identify HIV infection recency. The accurate identification of recent HIV infection continues to be an important research area and has implications for HIV prevention and treatment interventions. Longitudinal cohorts that follow HIV negative individuals over time are the current gold standard approach, but they are logistically challenging, time consuming and an expensive enterprise. Methods that utilize cross-sectional testing and biomarker information have become an affordable alternative to the longitudinal approach. These methods use well-characterized biological makers to differentiate between recent and established HIV infections. However, recent results have identified a number of limitations in serological based assays that are sensitive to the variability in immune responses modulated by HIV subtypes, viral load and antiretroviral therapy. Molecular methods that explore the dynamics between the timing of infection and viral evolution are now emerging as a promising approach. The combination of serological and molecular methods may provide a good solution to identify recent HIV infection in cross-sectional data. As part of this review, we present the advantages and limitations of serological and molecular based methods and their potential complementary role for the identification of HIV infection recency.

## 1. Introduction

Identification of HIV infection recency is crucial for the accurate estimation of HIV incidence, the monitoring of HIV spread [1,2,3], the understanding of HIV transmission dynamics [4,5,6,7] and the evaluation of prevention strategies [8]. The current “gold-standard” approach to identify the recency of infection involves the longitudinal follow-up and repeated testing of uninfected individuals [9,10,11]. However, implementation of this approach is logistically challenging, time consuming and expensive [8].

Methods that utilize cross-sectional testing have become an affordable alternative to the longitudinal cohort approach [12]. These methods rely on previously characterized biomarkers of recent infection [13,14,15]. Serology assays like Calypte Incidence Assay (BED) and Limiting Antigen Assay (LAg) classify recent infections based on markers of the host immune response to HIV, such as antibody levels, avidity, isotype and proportion [13,14,15,16]. These assays are generally cheaper, quicker and easier to implement at the population level than the longitudinal cohort approach [17]. However, recent results show that there are limitations in these assays as they are sensitive to variation in immune responses that are associated with different HIV subtypes, viral load levels and antiretroviral therapy (ART), among others.

Genomics-based assays and molecular methods that explore the dynamics between the timing of infection and viral evolution are now emerging as a promising approach [18,19]. These methods are based on the approximately linear genetic diversification of HIV over time [18,19,20,21,22] and make it possible to differentiate recent from established HIV infections [12,13,23,24,25,26,27]. In this manuscript, we specifically outline some of the recent advances in serology-based assays. We then examine a number of emerging molecular approaches. Finally, we discuss how serology and molecular based methods can be used together to estimate recent HIV infection from cross-sectional data.

## 2. Early Dynamics of HIV Infection

**Figure 1:**
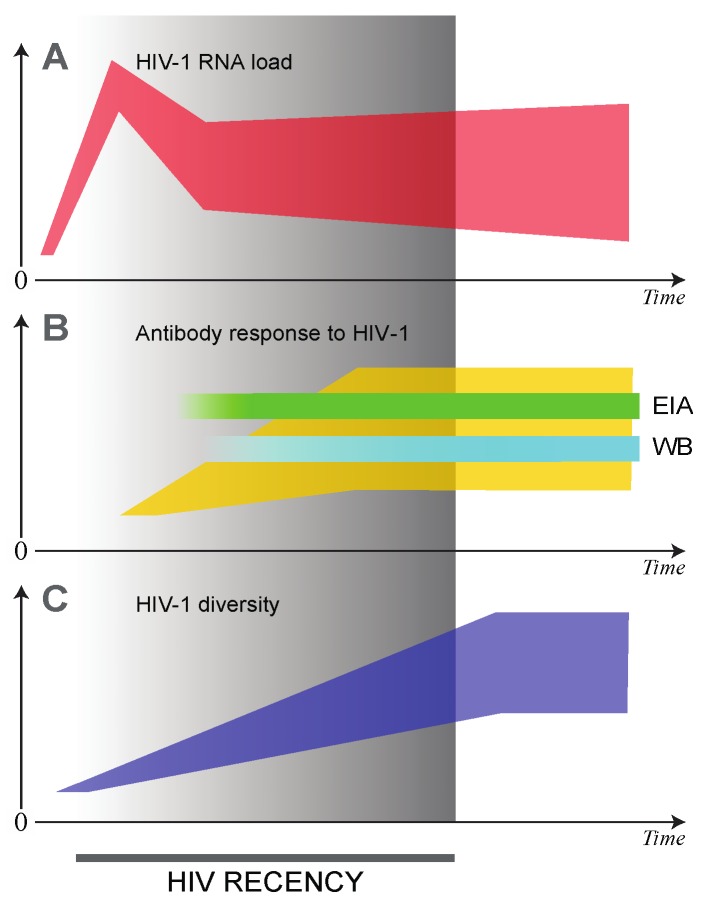
Markers of HIV recency: HIV-1 RNA load (**A**); antibody response to HIV-1 (**B**); and HIV-1 diversity (**C**). Heterogeneity of presented markers among HIV-infected individuals is highlighted by polygons (instead of single curves): red for HIV-1 RNA, yellow for cumulative anti-HIV antibodies, and blue for virus diversity. Time 0 indicates time of HIV transmission. The end of HIV recency period corresponds to approximately 12 months after HIV transmission. EIA: Enzyme immunoassay test for anti-HIV antibodies; WB: Western Blot.

HIV-1 RNA viral load is detectable within days after infection. This, coupled with the absence of HIV-1 antibodies during the early stages of infection, is a powerful marker for the identification of acutely infected individuals [28]. For this reason, it is possible to identify acute HIV infection by a combination of a negative HIV-1 antibody test and a positive viral load test (Figure 1) [28,29]. However, it is logistically difficult and expensive to identify acute HIV infections. This is because the acute phase of infection is very short (typically 2–4 weeks) [28,29,30]. In order to identify acute infections, one needs to follow a sero-negative cohort, which is repeatedly tested on a weekly basis until sero-converters are identified. On the other hand, recent HIV infection is considered to last between six and twelve months after infection [31]. This phase is the period when viral load set-point is established and antibody responses increase. Furthermore, HIV genetic diversity increases over time during this period (Figure 1).

## 3. Serological Tests of Recent HIV Infection

Several types of serological assays have recently been developed [14,17,23,24,32] and new biomarkers continue to be found [20]. These assays can differentiate between recently acquired and long-term infections based on (i) the increase in antibody titers [33]; (ii) the increase in the proportion of HIV-1 specific IgG antibodies [34]; (iii) the increase of antibody avidity [27,35]; and (iv) a combination of these markers [36]. HIV-infected individuals are classified as either recently or long-term infected if they fall above or below a well-defined threshold [26,37]. Serological assays have been reviewed extensively elsewhere [14,16,17,23,24]. We focus on describing the strengths and limitations of seven recently developed methods.

### 3.1. Less Sensitive Enzyme Immunoassays (LS-EIA)

The Less Sensitive Enzyme Immunoassays (LS-EIA), also known as the “detuned assay”, was one of the first serological assays to identify HIV infection recency [38]. This assay is based on the gradual increase in HIV-1 antibodies over time after sero-conversion. The underlying assumption is that recently infected individuals will have lower levels of HIV-1 antibodies compared to those with established infections. In this approach, specimens are serially tested with an enzyme immune-assay (EIA) that is diluted (“detuned”). A recent infection is identified when a specimen tests positive with a less diluted EIA but negative with a less sensitive EIA [38,39].

Commercial assays include the Abbott HAVAB (3A11 ELISA) and Avioq HIV-1 microelisa (formally marketed as BioMerieux Vironostika HIV microelisa) [17]. The 3A11 ELISA is no longer available commercially. These assays have been produced with antigens from subtype B, which is the most common HIV strain circulating in the USA and Europe. A number of studies have tried to evaluate these assays in the field with non-subtype B viruses [40,41]. However, non-B subtypes produced antibodies with reduced binding affinities, leading to an increase in the false-recency rate [42]. The LS-EIA assays are a potentially cost-effective and quick method to implement. However, they have to be adapted to different subtypes and evaluated on a larger scale across various geographical regions.

### 3.2. Proportion of HIV-1 Specific IgG Antibodies

Another frequently used serological assay is the BED capture enzyme immunoassay. This assay is commercialized as BED-CEIA by Calypte Biomedical, United States [34]. The BED assay estimates the proportion of HIV-1 specific IgG to the total IgG. Recent HIV infection is identified when the HIV specific IgG is lower than the total IgG. The assay uses peptides from immunodominant regions of gp41 glycoprotein of three different HIV-1 strains. These include (i) subtype B; (ii) circulating recombinant form (CRF_01AE); and (iii) subtype D. The use of three different strains allows the detection of recently infected individuals with different HIV-1 subtypes [34,43].

False-recent HIV infections are more likely to be identified in chronically infected individuals (*i.e.*, individuals in the late stage of HIV infection, elite controllers or individuals on ART) [14,26,44,45]. The performance of the BED assay can be improved by adding covariate information from clinical data or by adjusting for the false-recency rate. Its accuracy has also been found to vary by geography [46,47] and HIV-1 subtypes [48,49,50,51].

### 3.3. Antibody Avidity Assays

Another approach for identifying recent HIV infections is to investigate the quality of the antibody response. This can be done by measuring its avidity or strength of antibody-antigen binding. The Limiting Avidity assay (LAg) was the first assay to become commercially available to measure avidity. LAg measures the antibody binding to low concentrations of a multi-subtype peptides derived from an immunodominant region of gp41 [27,52]. In this assay, specimens are incubated with and without a chaotropic agent that disrupts antibody-antigen interactions. Antibodies normally become resistant to disruption over the course of the HIV infection. Those with low ratio avidity are indicative of recent HIV infection [53].

Avidity assays are normally more sensitive than the BED or detuned assays [52,53]. Nevertheless, some studies have shown that this assay can be affected by other pathogens, such as *Mycobacterium Tuberculosis* (TB). In addition, the assay misclassifies samples from individuals on ART with low viral load, and in people infected with HIV-1 subtypes D [48].

### 3.4. Anti-p24 IgG3

IgG3 is one of the second predominant subclasses in the antibody response towards HIV [54,55]. IgG3 isotypes to p24 antigen are present in early infection and then decline. This makes IgG3 an attractive biomarker for the identification of recent HIV infections [55,56,57], since high IgG3 levels are associated with a high HIV-1 viral load [58]. The HIV-1 Bio-Plex assay is one method that specifically measures the p24-specific IgG3 responses [59]. Although IgG3 has been observed to decline over time, about one-third of individuals exhibit relatively high IgG3 levels in the late stage of HIV-1 infection [59].

### 3.5. Inno-LIA HIV Adaptation

The Inno-LIA HIV-1/2 assay measures the increase in antibody-antigen reactivity following the seroconversion event [60]. The assay was first designed for the confirmation of an HIV diagnosis, and is similar to a western blot test [60]. The emergence of antibodies to various HIV-1 proteins at different time points after seroconversion is used to characterise the recency of infection [16,61]. The Inno-LIA assay detects antibodies to recombinant peptides of HIV-1 (p17, p24, p31, gp41 and gp120) and HIV-2 (gp36 and gp105). The intensity of the antibody-antigen bands is scored [61] and used to determine the recency of infection [16,17].

The Inno-LIA assay is advantageous because it can be used to confirm both an HIV diagnoses and a recent HIV infection. This assay can therefore significantly reduce costs. However, it can only detect a recent HIV infection within 36 to 67 days of the seroconversion date. The assay has not been evaluated in elite controllers, individuals receiving antiretroviral therapy and in individuals with a late stage of disease or AIDS [61].

### 3.6. Challenges Associated with the Application of Serological Assays in Cross-Sectional Data

There are limitations to the application of serological assays to determine recent HIV infection. Use of ART and low CD4+ cell counts can increase the false-recency rates. Low CD4+ cell counts are associated with low antibody responses (titers, proportion and avidity) [29]. Decreased antibody response is also associated with a low viral load level and a compromised immune function, which leads to the misclassification of long-term HIV survivors (*i.e.*, elite controllers or patients successfully responding to ART) as recently infected [61,62,63]. Co-infections also present additional challenges [16,24,46,48,64,65]. Lastly, false-recency rates have been shown to vary across HIV-1 strains and geographical regions [46,66].

The World Health Organization has established a Technical HIV Incidence Assay Working Group (HIVIWG) to provide guidelines and recommendations on how to use serological assays. These include adjusting certain parameters, such as the false-recency rate for a given geographical region [37,67]. In addition, there is a need to document the use of ART, viral load levels and CD4+ cell counts. However, detailed information may not always be obtained from surveillance programs in resource-limited settings. The HIVIWG hope that a new generation of avidity assays can overcome these challenges [36,68].

**Table 1:** Comparison of the molecular based approaches for determination of HIV recency.

Assay or Test Type or Recent Infection Algorithm	Brief Summary of the Assay or Test	Strengths	Limitations	References
High Resolution Melting Assay	Measures diversity of generated amplicons by using melting temperature of DNA duplexes.	Relatively inexpensive Does not require viral genotyping	PCR may be poor scalable in rural and resource constrained settings. Sensitive to indels that are common in HIV. Unable to distinguish between infections caused by single or multiple viral strains.	[69,70,71]
Counting Sequence Ambiguities	Ambiguous bases in the viral sequence indicate heterogeneous virus population. The number of ambiguous bases is small in recently infected individuals and increased overtime.	Can be cost effective if routine drug resistance testing is a part of clinical care	Not cost effective in resource limited settings. Can underestimate ambiguous positions. The impact of ART exposure is unknown and could be a serious concern. The number of ambiguous positions in the late stage of HIV infection could be reduced, which may lead to misclassifications.	[18,19,72,73]
Naïve Bayes Classifier (NBC)	Utilized the frequency of ambiguous sites together with CD4+ cell counts and any concurrent AIDS defining illness. The Bayesian probability framework estimates the probability of a patient to be in one of four stages of HIV infection.	High positive predictive value Can be retrospectively fitted to available genotypic and clinical data	Requires substantial validation. The method has been applied only once in HIV-1 subtype B settings.	[74]
Hamming Distance (HD)	The HD is a number that denotes the difference between two sequences of equal length. It is the simplest measure of HIV diversity. HD can measure the number of nucleotide differences between a pair of virus sequences. If applied to viral quasispecies from the host, HD can estimate the stage of HIV infection.	High sensitivity and specificity Simplicity	The HD approach has not been validated in long-term non-progressors, rapid progressors, and among ART-experienced individuals. It is unclear how indels and viral recombination can affect the HD estimates. Requires viral quasispecies. May have limited use in the resource-constrained settings.	[21,75]
Sequence Clustering Based Diversity Measure (SCBD)	Intra-cluster genetic diversity is used as the measure of time since infection. Inter-cluster diversity is used to determine whether there were multiple founder strains and the dot matrix incorporates information on indels and recombination.	Good accuracy High sensitivity and specificity	It is unclear how indels and viral recombination can affect the estimates. Is time consuming and expensive.	[76]
Multi-Assay Algorithms (MMA)	Results of serology-based test of recent infection combined with: Clinical data (e.g., CD4+ cell counts, HIV-1 RNA load, ART status) Measure of HIV diversity Combination of Assays can be optimized to increase accuracy.	Provides more accurate estimate of the HIV recency Reduces false recency	Not validated across HIV-1 subtypes and different populations. Requires clinical data (e.g., CD4+ cell counts, viral load count). Might be problematic logistically in resource-limited settings.	[69,77,78]

## 4. Molecular Tests of Recent HIV Infection

Most HIV infections are caused by a single transmission (*i.e.*, founder) virus, which results in an initial homogenous viral population that will diversify over time [79,80,81,82]. Upon transmission, the virus is able to diversify rapidly within the host due to immune response and the fact that HIV-1 Reverse Transcriptase enzyme is error prone. Virus genetic diversity increases in an approximately linear fashion for several years after infection, reaches a plateau, and declines in the late stage of infection [18]. Virus diversification in early HIV infection provides a strong rationale for identifying recent HIV infection [19,69,83,84]. Some methods, such as the high resolution melting assay (HMA), are able to estimate HIV recency without the use of a viral genotype [70]. However, the majority of molecular methods utilize viral sequences. Molecular methods that use sequences were developed for population [18,19,72] and single-genome or clonal sequencing [85]. Table 1 provides a summary of the strengths and limitations of molecular based methods. In this review, we describe five methods.

### 4.1. High Resolution Melting Assay

The high resolution-melting (HMA) assay is one of the simplest molecular methods. The assay uses the melting temperature of the DNA duplexes from amplicons. Multiple regions across the HIV-1 genome including *gag*, *pol* and *env* can be used [69,70,71] without the need to sequence them. HMA provides a single numeric score that reflects the level of diversity in the amplified region, which increases linearly over the course of the HIV infection [69]. There is concordance between the HMA score and viral diversity. This was obtained by next generation sequencing (NGS) and Shannon entropy analysis [86,87].

This assay is a relatively inexpensive technology that can be implemented in resource-limited settings that do not have access to sequencing infrastructure. For example, HMA has been successfully used in clinical trials in Uganda [88]. However, it has been shown to have limitations. For example, the assay is sensitive to insertions and deletions [89], which are common features of the HIV genome. Another limitation is its inability to distinguish between infections caused by single and multiple HIV strains. The HMA assay can be adapted to many HIV strains and could complement serological assays in resource-limited settings [90].

### 4.2. Sequence Ambiguities as a Marker of Recent HIV Infection

This approach is based on counting ambiguous nucleotide positions produced during population Sanger sequencing. Multiple nucleotides at the same position indicate that PCR amplification was performed from multiple templates and that the patient harbors heterogeneous viruses. The number of ambiguous positions increases as the viral population diversity grows over time [18,73]. For example, a linear increase in viral diversity within HIV-1 *pol* is associated with an increase of 0.2% ambiguous nucleotides per year [18]. A significant difference in the frequency of ambiguous sites has been observed among individuals with recent and established HIV infections in both HIV-1 subtype B and non-subtype B infections [18,73].

A number of different threshold values for the ambiguity index have been suggested. Anderson *et al.* [73] found that a threshold of 0.47% performed the best in discriminating recent (≤1 year) from established HIV infections. In that manuscript, the threshold of 0.47% returned a sensitivity of 74.5% and specificity of 87.2%. Brooks *et al.* [19] compared BED classification (≤6 months) with the ambiguity index using a threshold of 0.45%, which provided a sensitivity and specificity of 82.7% and 78.8%, respectively. They also found that varying the base-calling thresholds had little effect on the sensitivity and specificity. However, the two previously mentioned studies used different base calling strategies, PCR and sequencing methodologies, which makes it difficult to compare their results. One further limitation of the Brooks *et al.* paper is that the BED assay was used as the “gold standard” to identify recent HIV infections. As described earlier, this assay has been shown to have a high false-recency rate [14,26,44,45].

A limitation of this method is that it normally underestimates the number of ambiguous positions during Sanger sequencing [85]. Another limitation is the non-homogenous selective pressure of the immune system on HIV genetic regions, which makes it difficult to choose one unique threshold. Other limitations include the unknown impact of ART, the effect of low HIV-1 RNA in viremic/elite controllers and the reduced number of ambiguous positions in the late stage of HIV infection [91,92]. Lastly, HIV infection with multiple viruses can attenuate accuracy.

### 4.3. Naïve Bayes Classifiers

The naïve Bayes classifier is based on the observation that viral diversity increases in an approximately linear fashion over the course of HIV infection [80,91]. This method makes use of available clinical markers such as CD4+ cell counts over the course of HIV infection and any concurrent AIDS associated diagnosis [93]. Individuals are classified into one of four different stages of HIV infection. The first stage limits HIV infection to within one year, with two intermediate stages that include chronically infected individuals, and a fourth stage which corresponds to AIDS (as defined by a CD4+ < 200 copies/mL). A standard Bayesian model can be fitted to estimate the probability of an individual being in one of the four stages conditional on the frequency of ambiguous sites in the HIV-1 *pol* gene, the CD4+ cell count and a concurrent AIDS diagnosis. The naïve Bayes classifier was designed to have a high positive predictive value for identifying true recent HIV infections [74].

This method was successfully used to estimate the proportion of individuals at different stages of HIV infection in a large sequence cohort of HIV-1 subtype B infected men who have sex with men in the Detroit metropolitan area [74]. The study utilized HIV-1 *pol* sequences available through a routine drug resistance-screening program coupled with available clinical data to estimate prevalence, incidence and timing of HIV transmission. The authors found that individuals were eight times as infectious during the first year of HIV infection as compared with individuals who had established infections. They also found that 42% to 46% of the HIV transmissions came from individuals who were recently infected. This method has a great potential to understand how HIV recent infection can drive different HIV epidemics. However, it needs to be applied and evaluated in different geographic regions and with different HIV-1 strains.

### 4.4. Hamming Distances

The Hamming distance (HD) is a number that denotes the difference between two binary strings of equal length, *i.e.*, the number of positions at which the corresponding symbols are different. In relation to HIV diversity, HD can be used to measure the number of nucleotide differences between pairs of sequences. When applied to viral quasispecies, HD can be used to estimate the stage of HIV infection [21,75]. The HD of recent HIV infections, caused by a single founder virus, does not overlap with the HD from established HIV infections. However, HDs of infections resulting from multiple founder viruses can overlap, resulting in a misclassification as an established infection.

Park *et al.* devised a binary classification (recent *vs.* established infection) based on the tail characteristics of the Hamming distance distribution of HIV-1 *env* sequences [75]. An HD value that divides the lower 10% (Q10) of the HD frequency distribution from the upper 90% was shown to be able to distinguish a recent from an established HIV infection. The Q10 statistic was lower in recent HIV infections compared to chronic infections, and was shown to distinguish whether infections originated from a single or multiple founder strains. The authors also demonstrated a high sensitivity (97%) and specificity (100%) when applying the Q10 statistic.

This approach has recently been applied to high throughput pyrosequencing data and has the potential to be cost-effective for routine surveillance [94]. The test is yet to be validated in long-term non-progressors, rapid progressors and among ART-experienced patients. A further limitation is the potential impact of indels and recombination, particularly when using variable regions across the HIV-1 genome, such as *env*. The HD approach also does not take into account the nature of the evolutionary events. The assay relies on availability of viral quasispecies and might not be widely used in resource-limited settings without routine HIV genotyping infrastructure.

### 4.5. Sequence Clustering Based Diversity Measure

A sequence clustering based measure (SCBD) of HIV diversity has been proposed by Xia *et al.* [76]. The SCBD method determines HIV infection recency by using two principles. The first principle involves intra-patient clustering, which is defined as the closeness of viral quasispecies within an individual’s sample. An increase in intra-patient HIV diversity is correlated with the time since infection [76]. The second principle uses inter-patient HIV diversity, which is a measure of the presence of multiple founder viruses within an individual. The algorithm of the SCBD method first classifies an infection as recent if there is a low intra-patient diversity. If there is high inter-patient diversity, then the SCBD method will classify the sample as originating from a long-term infected individual. The purpose of the method is to reduce the misclassification of recent infections by using the inter-patient clustering measure.

In order to minimize the impact of indels on calculated diversity, the authors used a pairwise dot matrix alignment, which accounts for the nature of evolutionary events when calculating pairwise diversity. Xia *et al.* [76] proposed a SCBD cut-off of 1.0% based on the known evolutionary rate of the *env* gene [91]. The assay was tested on a dataset containing 398 incident and 163 chronic infection cases. It achieved an overall accuracy of 99.3%, with a sensitivity and specificity of 99.5% and 98.8%, respectively. The method overcomes the limitations of using only intra-patient diversity, since these do not account for the impact of indels and recombination on calculated diversity measures. The application of this method could be limited as the generation of HIV quasispecies are costly and labor intensive.

## 5. Multi-Assay Algorithms

HIV-1 subtype variability, heterogeneity of host immune responses and exposure to ART could impact the sensitivity and specificity of EIA assays. The combined use of different assays [36,67] could improve the accuracy of these methods to identify recent HIV infection. Recent HIV infection testing algorithms (RITA) can complement EIA assays by using clinical data (CD4+ cell counts, viral load data and ART use) to improve accuracy [12,68,77,81]. This approach helps to reduce false-recent rates caused by the natural or ART induced viral suppression [36,45]. Testing for traces of ART in the specimens could further reduce the misclassification of recent HIV infection [36,77,95].

Laeyendecker *et al.* [77] used a multi-assay algorithm (MAA) to test specimens with the BED assay. This procedure was then followed by viral load testing with a Bio-Rad avidity assay. Specimens with undetectable viral load were further tested for the presence of ART, improving the method to identify established cases of HIV infection [77].

An alternative MAA approach, which does not require viral load data, uses a Bio-Rad avidity (cut-off of 40% Avidity Index). It then confirms HIV infection recency using a LAg cut-off of normalised optical density at 2.8 [78]. This approach has a low false-recency rate, but the mean duration of HIV recency was reduced to 119 days and required larger sample sizes [78]. This MAA can be applied to dried blood spots making it an attractive method for resource-limited settings.

Another MAA begins by using CD4+ cell count information to exclude immuno-compromised individuals with AIDS [36,78]. Samples identified as recent HIV infections with the Bio-Rad avidity index assay were confirmed by the LAg assay. Recent infections classified by the LAg assay were then tested for viral load. Only specimens with detectable viral load were classified as recent infections regardless of ART exposure [78]. The mean duration of recency was identified as 146 days. One limitation is that a strong laboratory support is required for the implementation of MAAs that includes testing for viral load and ART exposure [78]. This support may not be fully available in resource-limited settings.

Requirement of fresh and whole blood CD4+ cell count may limit the applicability of MAAs. The additional resources required by the MAAs may not be justifiable for a marginal gain in precision. Novel MAA approaches have the potential to eliminate the need for CD4+ cell count and/or viral load information. One such approach, based on HIV diversity (HMA assay), was proposed by Cousins *et al.* [69]. This MAA tested specimens with BED and Bio-Rad avidity assays, and applied the HMA assay as a confirmatory test. The results were comparable to the MAA that included CD4+ cell count [69].

Well-designed and optimized MAAs will be able to improve the identification of HIV infection recency. However, this approach still needs to be evaluated across different HIV-1 subtypes and standardized [26].

## 6. Discussion

In this paper, we have provided a review of serological and molecular based methods to determine HIV infection recency. These methods provide a promising alternative to the standard longitudinal cohort approach, which is expensive, time consuming and difficult to implement. Methods that utilize cross-sectional biomarker information are likely to be more affordable and easier to implement in larger cohorts. Research has shown that serological based assays can detect recent HIV infections with a good accuracy at a substantially lower cost than longitudinal testing [14]. The new generation of antibodies avidity assays, for example, the LAg assay, has shown improved accuracy [68]. However, serological based assays are also sensitive to the variability in immune responses modulated by HIV clades, viral load levels and ART. Recent reviews of the serological based assays have concluded that there is an urgent need to develop new biomarkers while improving existing ones [20,96].

Molecular methods that use viral diversity have emerged as a novel and promising area of investigation and could complement existing serological based assays. Viral-based assays have a significantly lower misclassification rate than their serological counterparts, but are more expensive and difficult to implement [69,90,97]. However, recent advances in sequencing technology and the increasing availability of sequence data may enable the implementation of these assays in the field. The current assays are promising but require further validation and standardization for different settings, especially in sub-Saharan Africa where there are multiple circulating HIV subtypes and high prevalence. A Consortium for the Evaluation and Performance of HIV Incidence Assays (CEPHIA) has recently been established with the support from the Bill and Melinda Gates Foundation (BMGF) (Seattle, WA, USA). The aim of this consortium is to create a specimen repository to allow for an independent evaluation of assays [26] and algorithms.

Phylogenetic analysis has also emerged as a new tool. Combined with epidemiological and clinical data, phylogenies can be used to identify recently HIV infected individuals and to determine their contribution to the spread of local epidemics [74,98,99]. Volz *et al.* studied the dynamics of HIV transmission in Detroit and confirmed that a disproportionate fraction of viral transmissions originated from the very early stages of HIV infection [74]. They concluded that HIV transmissions could be reduced if recently infected individuals were identified and started on ART immediately [100].

It is likely that an optimal algorithm for the identification of HIV infection recency will be based on a combination of serological and molecular based assays. Low cost HIV diversity assays, such as HMA or the use of sequences already generated for drug resistance surveillance, could be implemented across resource-limited settings. Advances in high throughput sequencing could further improve affordability and scalability. The integration of clinical or epidemiological data with this approach, if available, could further improve the performance of MAAs. However, cost-effective studies will be needed to guide the implementation of MAAs across resource-limited settings. Implementing single genome amplification and NGS will be of further value in terms of addressing specific research questions related to viral linkage, sexual networks and the identification of sub-epidemics.

## 7. Conclusions

HIV infection recency can be estimated by a set of contemporary serological and molecular methods based using cross-sectional sampling. We expect that the methods reviewed here will further advance in the near future with the recently created WHO and BMGF consortiums. In addition, the usage of phylogenetic methods that take into account recent HIV infection will provide a better understanding of the transmission dynamics of HIV in sub-epidemics. These methods may replace longitudinal cohorts that are commonly used to evaluate HIV prevention and treatment interventions.
